# Bayesian optimisation with transfer learning for NASICON-type solid electrolytes for all-solid-state Li-metal batteries†

**DOI:** 10.1039/d2ra04539g

**Published:** 2022-10-26

**Authors:** Hiroko Fukuda, Shunya Kusakawa, Koki Nakano, Naoto Tanibata, Hayami Takeda, Masanobu Nakayama, Masayuki Karasuyama, Ichiro Takeuchi, Takaaki Natori, Yasuharu Ono

**Affiliations:** Department of Advanced Ceramics, Nagoya Institute of Technology Gokiso-cho, Showa-ku Nagoya Aichi 466-8555 Japan takeda.hayami@nitech.ac.jp; Department of Computer Science, Nagoya Institute of Technology Gokiso-cho, Showa-ku Nagoya Aichi 466-8555 Japan; RIKEN Center for Advanced Intelligence Project 1-4-1 Nihonbashi, Chuo-ku Tokyo 103-0027 Japan; Faculty of Engineering, Nagoya University Furo-cho, Chikusa-ku Nagoya Aichi 464-8601 Japan; TOAGOSEI CO., LTD, General Center of R&D 8, Showa-cho, Minato-ku Nagoya Aichi 455-0026 Japan

## Abstract

NASICON-type LiZr_2_(PO_4_)_3_ (LZP) has attracted significant attention as a solid oxide electrolyte for all-solid-state Li-ion or Li-metal batteries owing to its high Li-ion conductivity, usability in all-solid-state batteries, and electrochemical stability against Li metal. In this study, we aim to improve the Li-ion conductivity of Li-rich NASICON-type LZPs doped with CaO and SiO_2_, *i.e.*, Li_1+*x*+2*y*_Ca_*y*_Zr_2−*y*_Si_*x*_P_3−*x*_O_12_(0 ≤ *x* ≤ 0.3, 0 ≤ *y* ≤ 0.3) (LCZSP). Herein, a total of 49 compositions were synthesised, and their crystal structures, relative densities, and Li-ion conductivities were characterised experimentally. We confirmed the improvement in Li-ion conductivity by simultaneous replacement of Zr and P sites with Ca and Si ions, respectively. However, the intuition-derived determination of the composition exhibiting the highest Li-ion conductivity is technically difficult because the compositional dependence of the relative density and the crystalline phase of the sample is very complex. Bayesian optimisation (BO) was performed to efficiently discover the optimal composition that exhibited the highest Li-ion conductivity among the samples evaluated experimentally. We also optimised the composition of the LCZSP using multi-task Gaussian process regression after transferring prior knowledge of 47 compositions of Li_1+*x*+2*y*_Y_*x*_Ca_*y*_Zr_2−*x*−*y*_P_3_O_12_ (0 ≤ *x* ≤ 0.376, 0 ≤ *y* ≤ 0.376) (LYCZP), *i.e.*, BO with transfer learning. The present study successfully demonstrated that BO with transfer learning can search for optimal compositions two times as rapid as the conventional BO approach. This approach can be widely applicable for the optimisation of various functional materials as well as ionic conductors.

## Abstract 2

Demonstrate BO approaches to search for optimal composition with high ionic conductivity efficiently.

## Graphical abstract

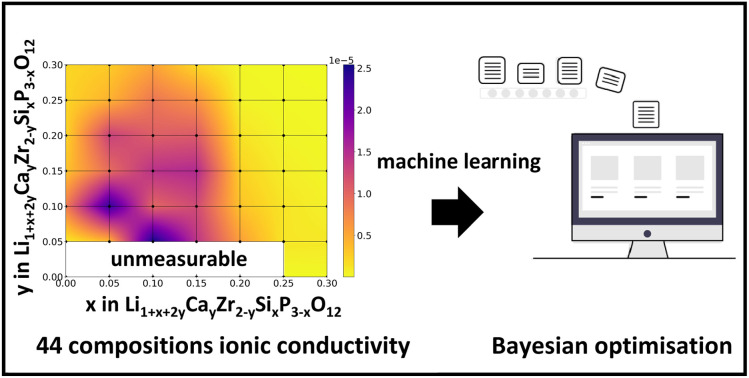

## Introduction

Since their commercialisation in 1991, Li-ion batteries (LIBs) have been used for powering portable electronic devices, such as smartphones and notebook computers, because of their high energy density and long cycle life.^1–4^ Currently, LIBs are used in large-scale storage devices, such as power sources for electrified vehicles.^5–10^ However, the use of organic liquid electrolytes in LIBs results in safety concerns such as leakage and explosion.^11–13^ Consequently, the development of all-solid-state Li secondary batteries using non-flammable inorganic solid electrolytes is currently attracting intense attention.^14–20^ Solid electrolytes have lower Li-ion conductivities than liquid electrolytes.^21,22^ Na superionic conductor (NASICON)-type oxides are considered to be promising solid electrolyte materials because of their high Li-ion conductivity.^23,24^ For example, NASICON-type Li_1.3_Al_0.3_Ti_1.7_(PO_4_)_3_ (LATP) exhibits a high bulk Li-ion conductivity of about 1 × 10^−3^ S cm^−1^ at room temperature.^25–27^ NASICON-type LiZr_2_(PO_4_)_3_ (LZP) family compounds are also highly conductive candidates for solid LIB electrolytes. For example, the bulk conductivity of Li_1.025_Y_0.025_Zr_0.975_(PO_4_)_3_ is 1.28 × 10^−4^ S cm^−1^ at 298 K.^28^ Although the bulk Li-ion conductivity of LZP family compounds is slightly lower than that of LATP, its phase stability in contact with Li metal is advantageous for practical application because of its high energy density as an anode material.^29–31^ Li *et al.* reported the stable charge–discharge operation of an all-solid-state Li-metal battery having a Li|LZP|LiFePO_4_ construction.^32^

**Fig. 1:**
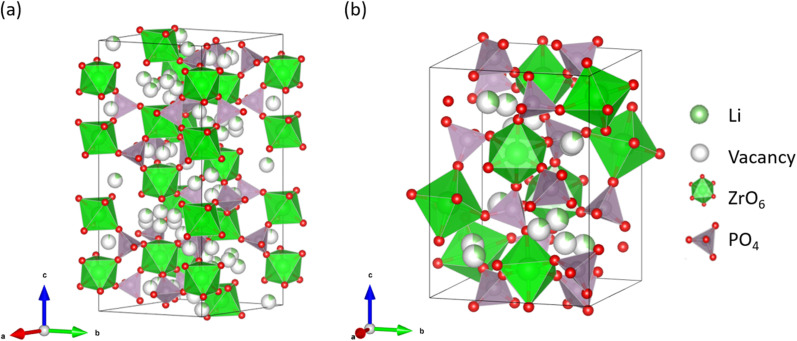
Two types of crystal structures of LiZr_2_(PO_4_)_3_: (a) α- and (b) β-phase structures.

LZP has four crystal phases that appear at different sintering temperatures, namely the α-, α′-, β-, and β′-phases,^33–35^ among which the α-phase exhibits the highest Li-ion conductivity of ∼10^−6^ S cm^−1^ at room temperature, which contains both bulk and grain-boundary contributions.^32^Fig. 1 shows the crystal structures of the α- and β-LZP phases.

In addition to the structure, the composition of LZPs influences their Li-ion conductivity. The Li-ion conductivity of NASICON-type solid electrolytes has been significantly increased by doping them with small amounts of low-valent metal oxides, which induce excess Li ions at the interstitial sites.^36–40^ For example, Li_1.2_Zr_1.9_Ca_0.1_(PO_4_)_3_, which was synthesised by partially replacing the Zr^4+^ of LZP with Ca^2+^, exhibited an improved total Li-ion conductivity of 4.9 × 10^−5^ S cm^−1^ at room temperature.^36^ An *ab initio* molecular dynamics investigation has ascribed the enhanced ionic conductivity of Li-rich NASICON-type electrolytes to the ‘pushing-out’ mechanism, whereby the additional Li ion displaces (‘pushes out’) a neighbouring Li ion due to strong coulombic repulsion, and the displaced Li ion repeats the process.^41^

However, the additional doping of Ca^2+^ ions decreases the Li-ion conductivity when *x* ≥ 0.2 in Li_1+2*x*_Zr_2−*x*_Ca_*x*_(PO_4_)_3_.^36^ This occurs due to the coulombic attraction between the Li interstitial site (
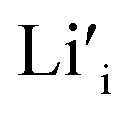
) and the Ca ion (
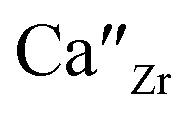
), which traps the Li ions.^42,43^ Therefore, when Ca is substituted into LZP, the effects of increasing the availability of Li ions and trapping Li ions around Ca are significant in terms of Li-ion conductivity. Additionally, Ca doping increases the degree of sintering, which may reduce the grain-boundary Li-ion conductivity.^39,44^ Therefore, optimisation of the LZP composition is important and effective for improving the Li-ion conduction performance. Recently, we exhaustively investigated the total Li-ion conductivities of Li_1+2*x*+*y*_Ca_*x*_Y_*y*_Zr_1−*x*−*y*_(PO_4_)_3_ (LCYZP), *i.e.*, LZP co-doped with CaO and Y_2_O_3_, and evaluated the co-doped composition that achieved the highest Li-ion conductivity.^39^ The compositional dependence of phase stability (α- or β-LZP phase), impurity phase concentration, and degree of sintering is highly complex; consequently, the intuitive approach toward compositional optimisation becomes difficult. Therefore, we demonstrated that Bayesian optimisation (BO) can analyse existing data effectively and suggest the upcoming sampling conditions, and thereby reduce the number of trial experiments.^45^ Likewise, Homma *et al.* optimised the Li-ion conductivity of the ternary Li_3_PO_4_–Li_3_BO_3_–Li_2_SO_4_ system using the BO approach.^46^ Suzuki *et al.* discovered the novel Li_3_Zn_0.65_Ge_4.35_O_10.85_ compound (10^−9^ to 10^−6^ S cm^−1^ at 300 K) from the Li_2_O–GeO_2_–P_2_O_5_ ternary system with the aid of machine-learning-based recommender system.^47–49^ Hence, informatics-derived approaches are beneficial for optimising the composition of solid electrolytes.

In this study, we investigated the Li-ion conductivities of 49 different compositions in the Li_1+*x*+2*y*_Ca_*y*_Zr_2−*y*_Si_*x*_P_3−*x*_O_12_(0 ≤ *x* ≤ 0.3, 0 ≤ *y* ≤ 0.3) (LCZSP) family, in which some of the Zr and P sites of LZP were replaced by Ca and Si ions, respectively. A few studies have been conducted on the replacement of the tetrahedral P site in the LZP structure; therefore, the present experimental results would add systematic knowledge that can guide the rational compositional optimisation of LZP family compounds.^50^ In addition, we demonstrated transfer learning for expediting the BO^51^ of the LCZSP composition by exploiting prior knowledge of LCYZPs. Conventional intuitive materials search relies principally on prior knowledge and expertise concerning the surrounding materials and phenomena. Similarly, the efficiency of the selection recommended by BO is expected to increase if transfer learning is appropriately utilised.^52,53^

## Experimental

In this study, 49 LCZSPs having the general formula Li_1+*x*+2*y*_Ca_*y*_Zr_2−*y*_Si_*x*_P_3−*x*_O_12_ (hereafter denoted as ‘Si_*x*_/Ca_*y*_’) with differing doping concentrations (0 ≤ *x* ≤ 0.3, 0 ≤ *y* ≤ 0.3) were synthesised by conventional solid-state reactions. Li_2_CO_3_ (99.9%; Kojundo Chemical Laboratory Co., Ltd), CaCO_3_ (99.5%; Kishida Chemical Co., Ltd), ZrO_2_ (99.9%; Kishida Chemical Co., Ltd), SiO_2_ (99.9%; Kojundo Chemical Laboratory Co., Ltd), and Zr(HPO_4_)·1.5H_2_O (Toagosei Co., Ltd) were used as the starting materials. Stoichiometric amounts of the reagents were thoroughly mixed with two times their combined mass of purified water (Kishida Chemical Co., Ltd) in an agate mortar. The mixture of reagents was dried and pressed into pellets at approximately 30 MPa and sintered in air at 200 °C for 6 h, then at 800 °C for 6 h, and finally at 1100 °C for 24 h. Thereafter, the samples were crushed and re-pelletised with a diameter of 10 mm and a thickness of approximately 1.8 mm by applying a pressure of approximately 130 MPa, followed by sintering in air at 1200 °C for 4 h. The apparent densities of the pellets were evaluated by measuring their geometrical diameters and thicknesses. The theoretical density was calculated by referring to the structural data reported by Petit *et al.*^54^ for the calculation of relative densities. Morphological and elemental analyses were performed by scanning electron microscopy (SEM, JSM-6460LV, JEOL, Japan) in combination with energy-dispersive X-ray spectroscopy (EDX). Powder X-ray diffraction (XRD, Rigaku Mini Flex 600, Japan) patterns recorded with Cu Kα radiation in air at room temperature were analysed to investigate the phase formation. To measure the ionic conductivity, the sintered pellets were polished using 2000-grit emery paper, and Au metal was sputtered to function as an electrode on both sides of the pellets. The Li-ion conductivities of the pellets were measured at 30, 60, and 90 °C *via* a typical complex AC impedance method in the frequency range of 1 Hz to 1 MHz using a BioLogic VMP-300 potentiostat. The activation energy (*E*_a_) for Li-ion conductivity was calculated from the Arrhenius plot of the total Li-ion conductivity. The impedance data were fitted by a complex non-linear least squares regression implemented in the ZView software. To evaluate the stability of Ca- and Si-substituted LZPs in contact with Li metal, the galvanostatic cycle performance was evaluated using the Li|LCZSP(Si_0.1_/Ca_0.05_)|Li symmetric cell under Ar atmosphere. The measurements were conducted at 80 °C in an Ar-filled glove box. The cut-off voltage and current density were set to 5 V and 50 μA cm^−2^, respectively. BO was performed to determine the optimal composition that results in the highest ionic conductivity among the experimentally evaluated samples. Exhaustive experimental data have been acquired for the entire compositional range used in this study; consequently, the present BO is for demonstrational purpose only. The conductivities of 44 samples at 30 °C were considered as variables and the values of *x* and *y* in Si_*x*_/Ca_*y*_ were considered as descriptors for BO. The random search and the search with BO were performed with 44 trials each. The expected improvement (EI)^55^ strategy was used to determine the acquisition function, and each candidate point was observed once. Furthermore, to implement transfer learning for BO, the similarity between the data sets was incorporated into the Gaussian process (GP) as a kernel function. The automatic relevance determination (ARD) Gaussian kernel was used for the GP, and the kernel parameters, inter-task covariance matrix,^56^ and noise variance of the GP were determined by maximising the marginal likelihood for each experiment. The previously reported conductivities of 47 LZP samples with Zr partially replaced by Y and Ca ions at Zr sites (Li_1+*x*+2*y*_Y_*x*_Ca_*y*_Zr_2−*x*−*y*_P_3_O_12_, 0 ≤ *x* ≤ 0.376, 0 ≤ *y* ≤ 0.376) (LYCZP) were used as the training data, *i.e.*, prior knowledge, to evaluate the acceleration of the BO scheme for the present 44 LCZSP samples. The algorithm was run for all 44 patterns of initial points, with one LCZSP data point provided as an initial point. Using this model, the LYCZP data can be used for more efficient compositional optimisation.

## Results and discussion

**Fig. 2:**
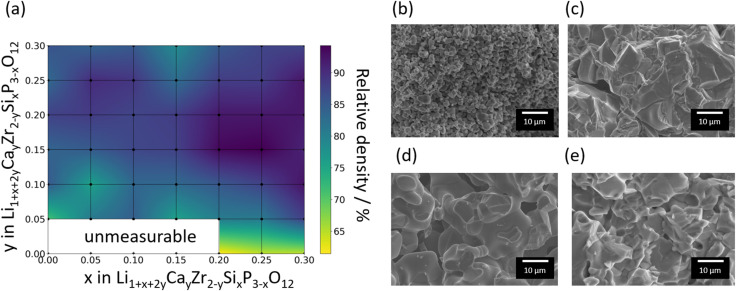
(a) Compositional dependence of the relative density of sintered Si_*x*_/Ca_*y*_ pellets. SEM images of the fracture surfaces of sintered samples of (b) Si_0.3_/Ca_0_, (c) Si_0.3_/Ca_0.1_, (d) Si_0_/Ca_0.05_, and (e) Si_0.1_/Ca_0.05_.

Fig. 2a shows the relative densities of the 44 sintered pellets at room temperature, where the theoretical density is assumed to be 3.13 g cm^−3^. The relative densities and ionic conductivities of four samples, Si_0_/Ca_0_, Si_0.05_/Ca_0_, Si_0.1_/Ca_0_, and Si_0.15_/Ca_0_, could not be measured due to insufficient densification of pellets. The relative densities of the samples without Ca substitution were lower than 65%; however, the relative density increased significantly with increasing Ca substitution. Fig. 2b and c shows SEM images of the fracture surfaces of the samples without and with Ca substitution, respectively, at constant Si substitution. The shape of each particle was clearly visible for the sample Si_0.3_/Ca_0_, as shown in Fig. 2b. Consequently, its grain boundaries were readily identified. In contrast, the sintered body of Si_0.3_/Ca_0.1_ was homogeneously structured because of grain growth, as shown in Fig. 2c. Evidently, Ca substitution promotes grain growth, thereby increasing the relative density, as shown in Fig. 2a and reported previously.^39^ The sintering effect of Si doping into P ions was also investigated by comparing SEM images of the fracture surfaces of the samples without and with Si doping (Fig. 2d and e). A slight improvement in the particle-to-particle bonding at the neck was observed, whereas the grain growth effect of Si doping was insignificant, as was the increase in relative density.

**Fig. 3:**
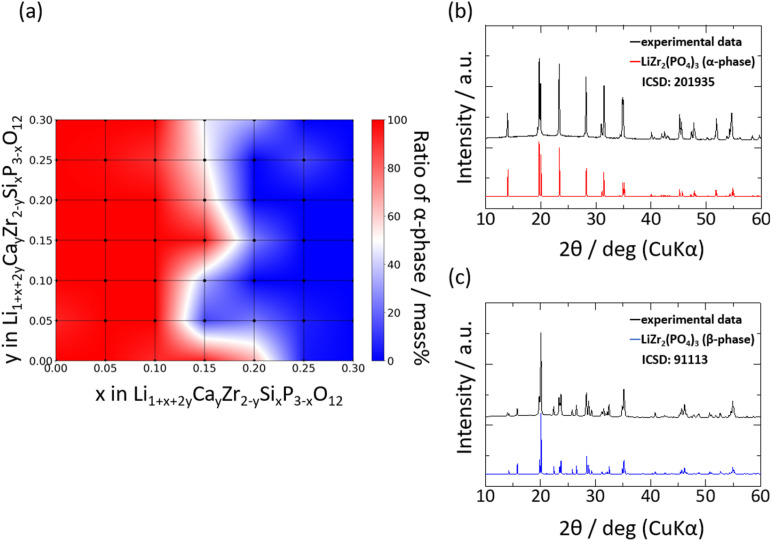
(a) Weight ratio of α- and α′-phases of the as-synthesised Si_*x*_/Ca_*y*_ pellets. The blue areas in the heat map are composed of β-phases. XRD profiles of selected compositions, (b) Si_0.1_/Ca_0.05_ and (c) Si_0.3_/Ca_0.15_, compared with the calculated profiles of α-phase LiZr_2_(PO_4_)_3_ (ICSD: 201935) and β-phase LiZr_2_(PO_4_)_3_ (ICSD: 91113).

Fig. 3 shows the XRD patterns of selected samples. The XRD patterns indicated that the main phase of the samples was one of four types of polymorph structures of LZP, namely the α- (ICSD: 201935), α′- (ICSD: 89456), β- (ICSD: 91113), and β′- (ICSD: 91112) phases. In addition to the LZP-derived peaks, several samples exhibited additional peaks attributed to impurity phases such as ZrO_2_ and CaCO_3_. The weight fractions of the phases identified by analysing the XRD patterns are shown as a heat map in Fig. 3a using the PDXL software (Rigaku).

The α- and α′-phases dominated at low fractions of Si (*x* ≤ 0.2), whereas the β- and β′-phases dominated at *x* > 0.2 in Si_*x*_/Ca_*y*_. This transition to the β- and β′-phases may be ascribed to an elevation in the phase transition temperature, whereby the β- or β′-phase stabilised upon increasing the weight fraction of Si in the lattice. EDX analyses were performed on selected samples to evaluate the distribution of Ca or Si dopants in the sintered body, and the elemental maps are shown in Fig. S1.† Fig. S1a–c† depicts the distribution of Ca ions in the sintered bodies. The distribution of Ca ions was uniform at lower Ca contents, whereas the condensation of Ca ions in the vicinity of the grain boundary was visible for highly Ca-doped samples, as reported previously.^36^ In contrast, Si ions were distributed uniformly irrespective of the number of Si ions in the lattice. Therefore, the solubility limit of Ca ions is lower than that of Si ions. Fig. S1d and e† depicts the abundance of Si.

**Fig. 4:**
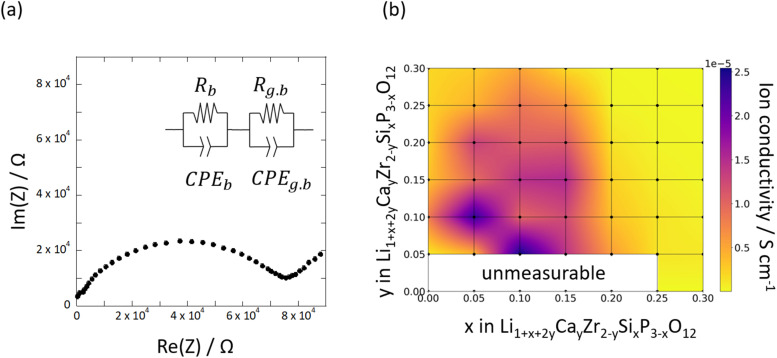
(a) Typical complex AC impedance plot of Si_0.2_/Ca_0.1_ at 30 °C and (b) compositional dependence of the measured Li-ion conductivity of synthesised Si_*x*_/Ca_*y*_ pellets.

Fig. 4a shows the Nyquist plots derived from the complex AC impedance measurement of Si_0.2_/Ca_0.1_ at 30 °C. The Nyquist plots consisted of a single semicircle in the high-frequency region and a low-frequency impedance spike. The high-frequency semi-circles were flattened, indicating the inclusion of multiple resistance components. Because the capacitance of this semicircle was in the range of 10^−10^ to 10^−11^ pF, we inferred that the bulk resistance and grain-boundary resistance overlap. Therefore, the Li-ion conductivity obtained in this study corresponds to the total resistance of bulk and grain boundaries. Fig. 4b shows the compositional dependence of Li-ion conductivities measured at 30 °C as a heat map. The highest ionic conductivity was exhibited by Si_0.1_/Ca_0.05_ in the temperature range of 30–90 °C (*e.g.*, 2.56 × 10^−5^ S cm^−1^ at 30 °C, as shown in Fig. 4b). The highest ionic conductivity obtained in the present study was 2.56 × 10^−5^ S cm^−1^, which is almost equal to or slightly lower than the values obtained in previous studies. For example, the ionic conductivities of Ca-,^44^ Y-,^28^ and Sr-doped^57^ LZPs at room temperature were reported as 1.2 × 10^−4^, 1.28 × 10^−4^, and 3.44 × 10^−5^ S cm^−1^, respectively. However, it must be noted that these reported values refer to the bulk ionic conductivity, while the values reported here refer to the total conductivity (bulk and grain-boundary conductivities). Therefore, the present results are comparable to the highest ionic conductivities of LZP family compounds in the literature. The highest ionic conductivity was not observed at the edge of the compositional range but appeared at an intermediate composition, which indicates a trade-off between the effects of Ca and Si doping. In general, the Si-rich samples exhibited lower ionic conductivities of Li, which corresponds to the stabilisation of the β-phase at *x* > 0.2. Evidently, β-phase formation is the primary reason for the decrease in ionic conductivity with increasing Si doping. The α-phase was stable even for highly Ca-doped compositions, and the ionic conductivity decreased with an increase in the Ca content (*y* > 0.05). This can be attributed to the formation of impurity phases at the grain boundaries due to Ca substitution, which increases the grain-boundary resistance, according to the EDX maps presented in Fig. S1.† Furthermore, the trapping of Li ions by Ca^2+^ ions at Zr^4+^ sites may reduce the bulk ionic conductivity, as reported in our previous computational study.^41^ Activation energies were evaluated using Arrhenius plots, as shown in Fig. S2a and b† shows a heat map of the activation energy for the Li-ion conductivities of Si_*x*_/Ca_*y*_ pellets.

In the heat map, the activation energy is relatively high for compositions at which the β-phase is stable (0.2 ≤ *x*). The activation energies tend to reduce in the composition ranges of 0.05 ≤ *x* ≤ 0.15 and 0.10 ≤ *y* ≤ 0.15, where the α-phase stabilises, indicating that co-doping with CaO and SiO_2_ is effective at increasing the Li-ion conductivity, as shown in Fig. 4b. The optimised composition is Si_0.1_/Ca_0.05_, at which the activation energy is 0.378 eV. This composition is optimal because of the reduction of the trapping effect caused by using a lower-valent defect, (
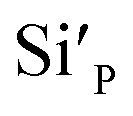
) and the increase in the degree of sintering caused by CaO doping. A target cell consisting of Li|LCZSP(Si_0.1_/Ca_0.05_)|Li was constructed for the sample, and the DC galvanostatic cyclic voltage profile was evaluated (Fig. S3†). No short-circuit behaviour was observed during the measurement (∼110 h), suggesting that LCZSP was relatively stable during the reduction reaction with Li metal. LZP is known to react with metallic Li to produce small amounts of Li_8_ZrO_6_ and Li_3_P.^32,41^ We hypothesise that the small amounts of impurities mentioned above were formed in this system as well.

**Fig. 5:**
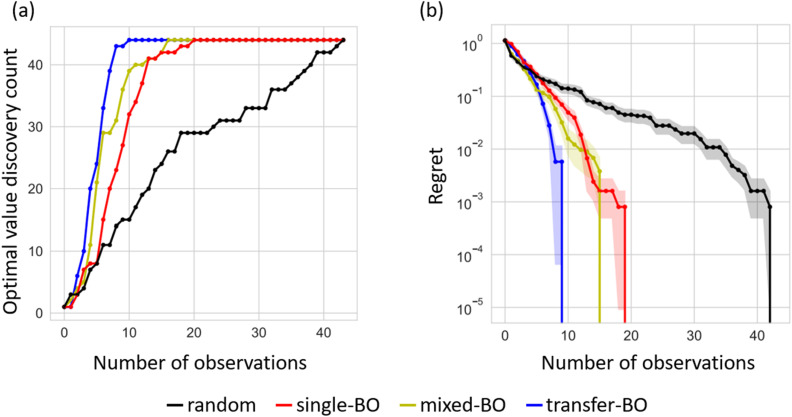
Performance comparison among three types of Bayesian optimisation (BO) approaches and the random search approach for the optimisation of Li-ion conductivity at 30 °C. The red, yellow, blue, and black lines indicate single-BO, mixed-BO, transfer-BO, and random search, respectively. Panel (a) shows the discovery rate for the highest Li-ion conductivity, and panel (b) plots the regret, which is the difference between the optimal value and the highest ionic conductivity among those of the observed compounds. Note that the discovery rate and the regret are averaged against 44 search trials.

Using the exhaustive data of Li-ion conductivity we collected and shown in Fig. 4b, BO was conducted to demonstrate the efficiency of discovering optimised compositions. Fig. 5a plots the rate of discovery of the compound having the highest ionic conductivity. Fig. 5b plots the difference between the highest ionic conductivity among the measured data and that among all the candidate compositions (including those of unmeasured samples), which is termed as ‘regret’ as a function of the number of experiments for LCZSP compounds. The red line corresponds to a BO using only the present LCZSP data (single-BO), which exhibited a quicker discovery than random sampling (black line).

Specifically, the discovery rate reached 100% after the 20th sampling among 44 candidates, indicating that the efficiency of single-BO is approximately two times that of random sampling. The yellow line corresponds to a BO approach starting with 47 LCYZP datasets derived from our previous study (mixed-BO) and one randomly selected LCZSP composition, and the standard BO scheme was followed. In total, 44 BO evaluations were performed. The optimisation process of mixed-BO exhibited a higher discovery rate and lower regret compared with those of single-BO, demonstrating that prior knowledge about LCYZPs was beneficial for the optimisation process. We confirmed that the optimisation performance of the transfer-BO approach exceeded those of single-BO and mixed-BO, with 10 transfer-BO samplings attaining a discovery rate of 100% for optimised composition, which is two times as rapid as single-BO. We infer that the quicker optimisation of mixed- and transfer-BO than that of single-BO originated from the reduction of exploration steps, which preferentially select sample(s) having higher variance, since prior knowledge of LCYZP helps reduce such variance in the prediction even in the early stages of BO. Accordingly, the present BO studies successfully demonstrate the acceleration of functional materials search by transfer learning based on past experimental data. Note that transfer-BO, in principle, will avoid falling into performance degradation by biasing the past knowledge through multiple-output GP regression.

## Conclusions

In this study, we synthesised 49 different compositions of the Li_1+*x*+2*y*_Ca_*y*_Zr_2−*y*_Si_*x*_P_3−*y*_O_12_ (0 ≤ *x* ≤ 0.3, 0 ≤ *y* ≤ 0.3) family, in which Ca and Si ions were substituted at Zr and P sites of LiZr_2_(PO_4_)_3_, respectively, and measured their Li-ion conductivities. The highest Li-ion conductivity of 2.56 × 10^−5^ S cm^−1^ at 30 °C was observed for the composition Si_0.05_/Ca_0.1_, proving that the co-doping of Si and Ca ions into LZP is effective at optimising the ionic conductivity. The compositional dependence of the measured Li-ion conductivity is very complex because the phase stability of the four types of polymorphs and the degree of sintering of the samples exhibit differing trends, increasing the difficulty of conventional intuition-driven materials optimisation. We demonstrated efficient materials optimisation by machine-learning-driven BO (single-BO), whereby 20 samplings among 44 samples were sufficient to discover the optimal composition. Transfer-BO for 44 LCZSP compositions using pre-inputted datasets of 47 LYCZP compositions and multi-task GP prediction exhibited further enhanced efficiency. Approximately 10 samplings were sufficient to discover the composition having the highest Li-ion conductivity at 30 °C; therefore, transfer-BO was approximately four times as efficient as random search. The as-developed transfer-BO approach may be applicable to more general materials optimisation applications including compositional optimisation and processing parameter optimisation. In other words, dead-stored materials data may expedite the time-consuming optimisation process.

## Author contributions

H. F., H. T., M. N., and Y. O. conceived and directed the project. H. F., and H. T. conducted the experimental synthesis and property measurements. H. F., S. K., K. N., M. N., M. K., and I. T. performed the Bayesian optimisations. The manuscript was written through the contributions of all authors. All authors have given approval to the final version of the manuscript.

## Conflicts of interest

There are no conflicts to declare.

## Supplementary Material

RA-012-D2RA04539G-s001
