# Supplementary material for: Bayesian optimisation with transfer learning for NASICON-type solid electrolytes for all-solid-state Li-metal batteries
Source: RSC Adv. 2022 Oct 26;12(47):30696–703. doi: 10.1039/d2ra04539g (PMC9597857; doi:10.1039/d2ra04539g)
Supplement: RA-012-D2RA04539G-s001 [file RA-012-D2RA04539G-s001.pdf]

Electronic Supplementary Material (ESI) for RSC Advances.
This journal is © The Royal Society of Chemistry 2022

Supplementary Information

Bayesian optimisation with transfer learning for NASICON-type solid electrolytes for all-solid-state Li metal batteries

Hiroko Fukuda[a], Shunya Kusakawa[b], Koki Nakano[a], Naoto Tanibata[a], Hayami Takeda*[a], Masanobu Nakayama[a], Masayuki Karasuyama[a], Ichiro Takeuchi[b,c,d], Takaaki Natori[e] and Yasuharu Ono[e]

[a] *Department of Advanced Ceramics, Nagoya Institute of Technology, Gokiso-cho, Showa-ku, Nagoya, Aichi 466-8555, Japan*

[b] *Department of Computer Science, Nagoya Institute of Technology, Gokiso-cho, Showa-ku, Nagoya, Aichi 466-8555, Japan*

[c] *RIKEN Center for Advanced Intelligence Project, 1-4-1 Nihonbashi, Chuo-ku, Tokyo 103-0027, Japan*

[d] *Faculty of Engineering, Nagoya University, Furo-cho, Chikusa-ku, Nagoya, Aichi 464-8601, Japan*

[e] *TOAGOSEI CO., LTD., General Center of R&D, 8, Showa-cho, Minato-ku, Nagoya, Aichi 455-0026, Japan*

*Corresponding author. E-mail: takeda.hayami@nitech.ac.jp (Hayami Takeda)

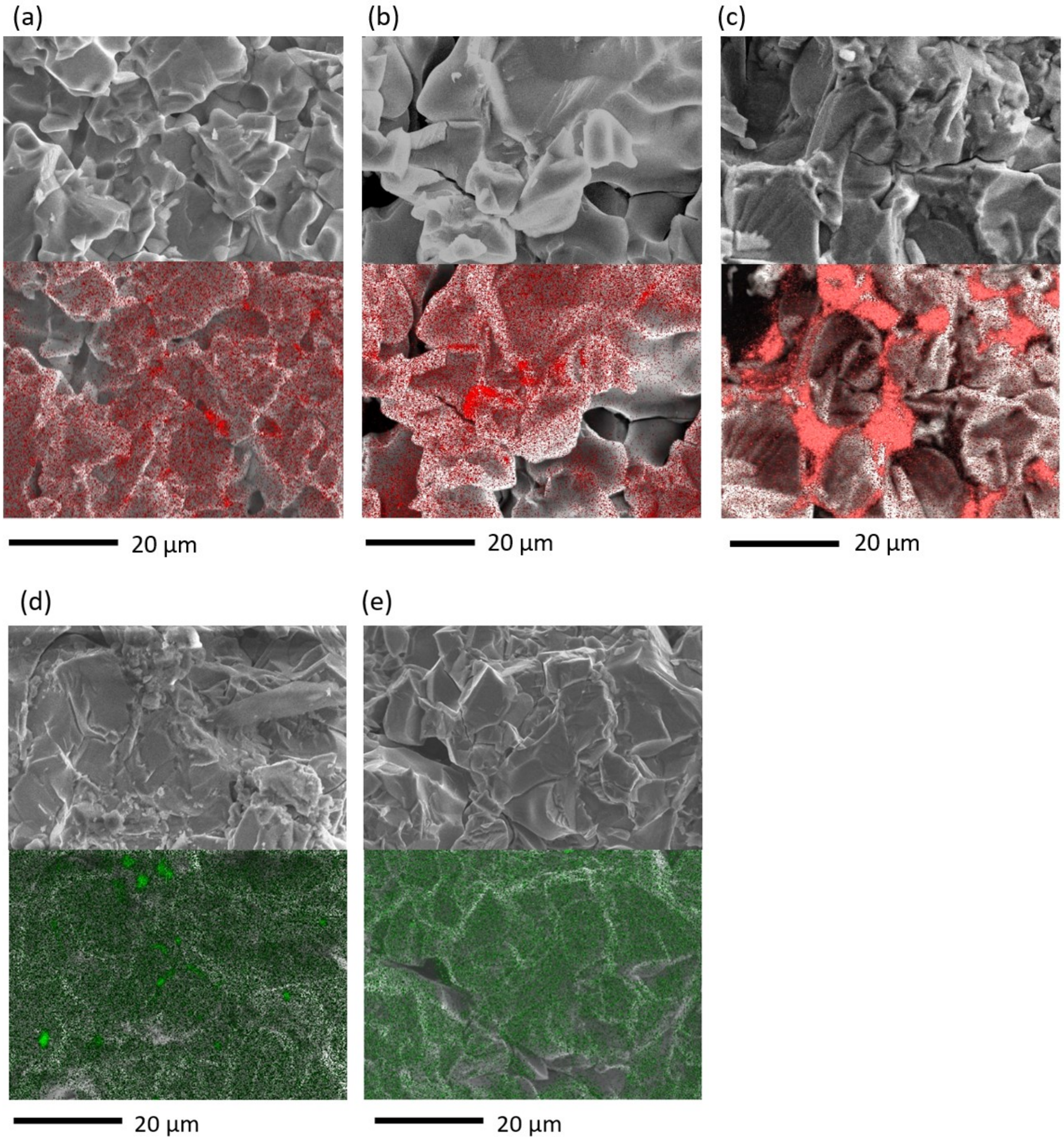

Fig. S1 EDX elemental maps of calcium (red) and silicon (green) in the fracture surfaces of (a) $Si_{0.1}/Ca_{0.05}$, (b) $Si_{0.1}/Ca_{0.1}$, (c) $Si_{0.1}/Ca_{0.3}$, (d) $Si_{0.2}/Ca_{0.1}$, and (e) $Si_{0.3}/Ca_{0.1}$.

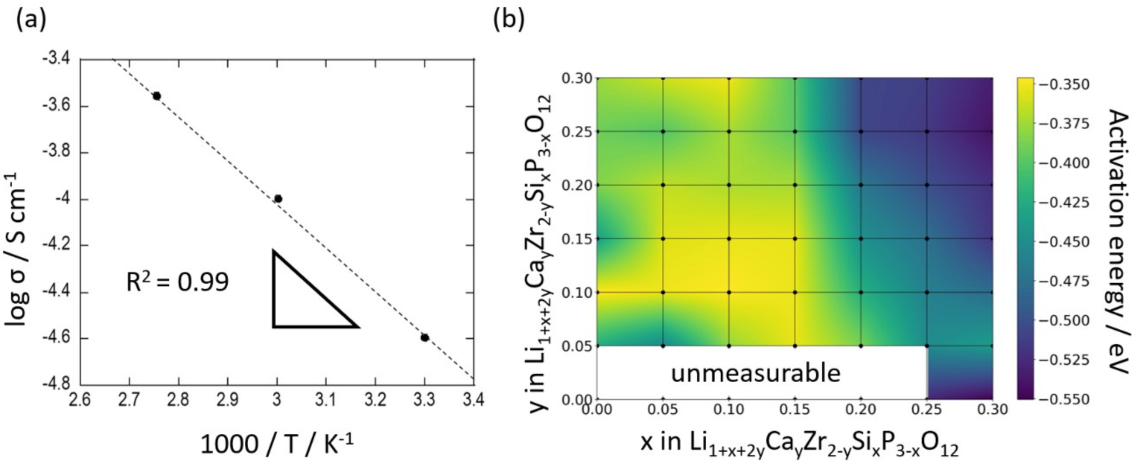

Fig. S2 (a) Arrhenius plot of the Li-ion conductivity of $Si_{0.1}/Ca_{0.05}$. (b) Composition dependence of the activation energy for the as-synthesised $Si_{\boldsymbol{x}}/Ca_{\boldsymbol{y}}$ pellets.

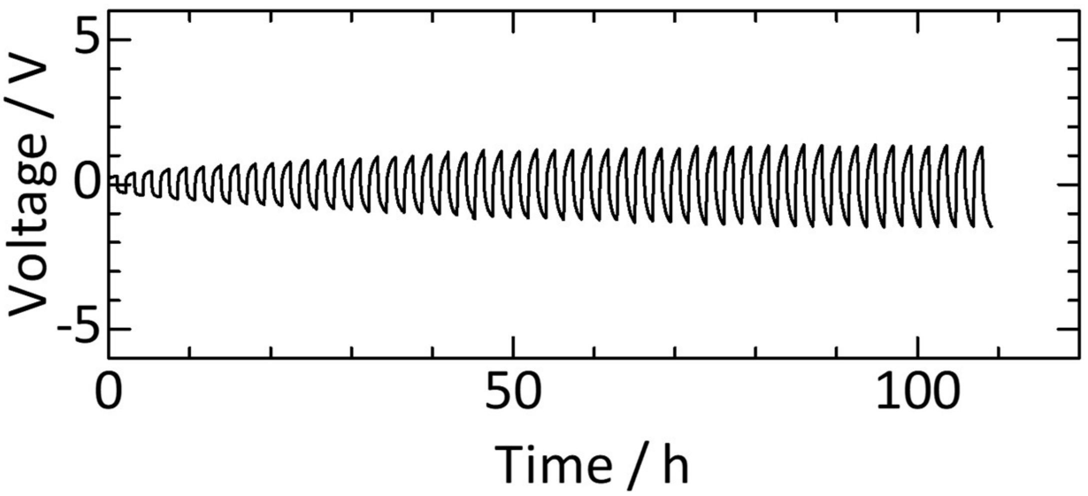

Fig. S3 Galvanostatic cycle measurement of the Li | $Li_{1.2}Ca_{0.05}Zr_{1.95}Si_{0.1}P_{2.9}O_{12}$ | Li symmetric cell over a period of 110 h at a current density of 50 μA cm^{-2}.
